# A New Method for Discovering Disease-Specific MiRNA-Target Regulatory Networks

**DOI:** 10.1371/journal.pone.0122473

**Published:** 2015-04-07

**Authors:** Miriam Baglioni, Francesco Russo, Filippo Geraci, Milena Rizzo, Giuseppe Rainaldi, Marco Pellegrini

**Affiliations:** 1 Institute of Informatics and Telematics (IIT), National Research Council (CNR), Via G. Moruzzi 1, 56124, Pisa, Italy; 2 Laboratory of Integrative Systems Medicine (LISM), Institute of Informatics and Telematics (IIT) and Institute of Clinical Physiology (IFC), National Research Council (CNR), Via G. Moruzzi 1, 56124, Pisa, Italy; 3 Department of Computer Science, University of Pisa, Largo Bruno Pontecorvo 3, 56127, Pisa, Italy; 4 Institute of Clinical Physiology (IFC), National Research Council (CNR), Via G. Moruzzi 1, 56124, Pisa, Italy; Weizmann Institute of Science, ISRAEL

## Abstract

Genes and their expression regulation are among the key factors in the comprehension of the genesis and development of complex diseases. In this context, microRNAs (miRNAs) are post-transcriptional regulators that play an important role in gene expression since they are frequently deregulated in pathologies like cardiovascular disease and cancer. In vitro validation of miRNA - targets regulation is often too expensive and time consuming to be carried out for every possible alternative. As a result, a tool able to provide some criteria to prioritize trials is becoming a pressing need. Moreover, before planning in vitro experiments, the scientist needs to evaluate the miRNA-target genes interaction network. In this paper we describe the *miRable* method whose purpose is to identify new potentially relevant genes and their interaction networks associate to a specific pathology. To achieve this goal *miRable* follows a system biology approach integrating together general-purpose medical knowledge (literature, Protein-Protein Interaction networks, prediction tools) and pathology specific data (gene expression data). A case study on Prostate Cancer has shown that *miRable* is able to: 1) find new potential miRNA-targets pairs, 2) highlight novel genes potentially involved in a disease but never or little studied before, 3) reconstruct all possible regulatory subnetworks starting from the literature to expand the knowledge on the regulation of miRNA regulatory mechanisms.

## Introduction

Nowadays a huge amount of biological data (e.g. gene and protein expression data) is available to scientists to be used to dissect the complexity of a disease. However, extracting useful information from biological databases is a complex task, in fact, it has to be understood and mined searching for the sparkling gems. This is a though job to do without the help of tools able to identify the most promising options. This task becomes even harder when we consider gene regulation and miRNAs as post-transcriptional regulators. miRNAs are small non-coding RNAs that negatively regulate gene expression at post-transcriptional level, usually binding the 3’-UTR of mRNAs [1]. Several tools for the prediction of miRNA-mRNA interactions are available. However they generally predict whether a gene is a target for a miRNA analyzing only the sequence (TargetScan [2], miRanda [3] and Pita [4]). Other tools are based on sequence and gene expression data (Magia2 [5]). Methods based only on the analysis of the sequence tend to return a large number of false positive targets. Computational methods predict hundreds of thousands target mRNAs per miRNA [6]. According to several studies, the expected rate of false positive returned by these predictors can range from 24% to 70% [7–9]. Methods based on the integration of mRNA and miRNA expressions can improve the prediction accuracy, even if they do not take into account the importance of each gene in relation to its disease-specific regulatory network.

In this work we attempt to overcome these problems by integrating disease-specific knowledge collected from the literature as well as the mRNA—miRNA expression data and the protein-protein interaction networks. Protein-Protein Interaction networks play an important role in the identification of disease associated genes, and can be further explored in order to identify disease related subnetworks (see [10] and references therein).

In this paper we describe *miRable*, a new method that takes into account the disease-specific context, mRNA—miRNA expression data and protein-protein interaction networks to provide a landscape of the complex miRNA-gene regulatory networks that are at the root of a specific pathology. As a case study, we tested our method on Prostate Cancer (PCa) discovering a promising gene and two miRNAs still unstudied in conjunction with this pathology.

## Materials and Methods

**Fig 1:**
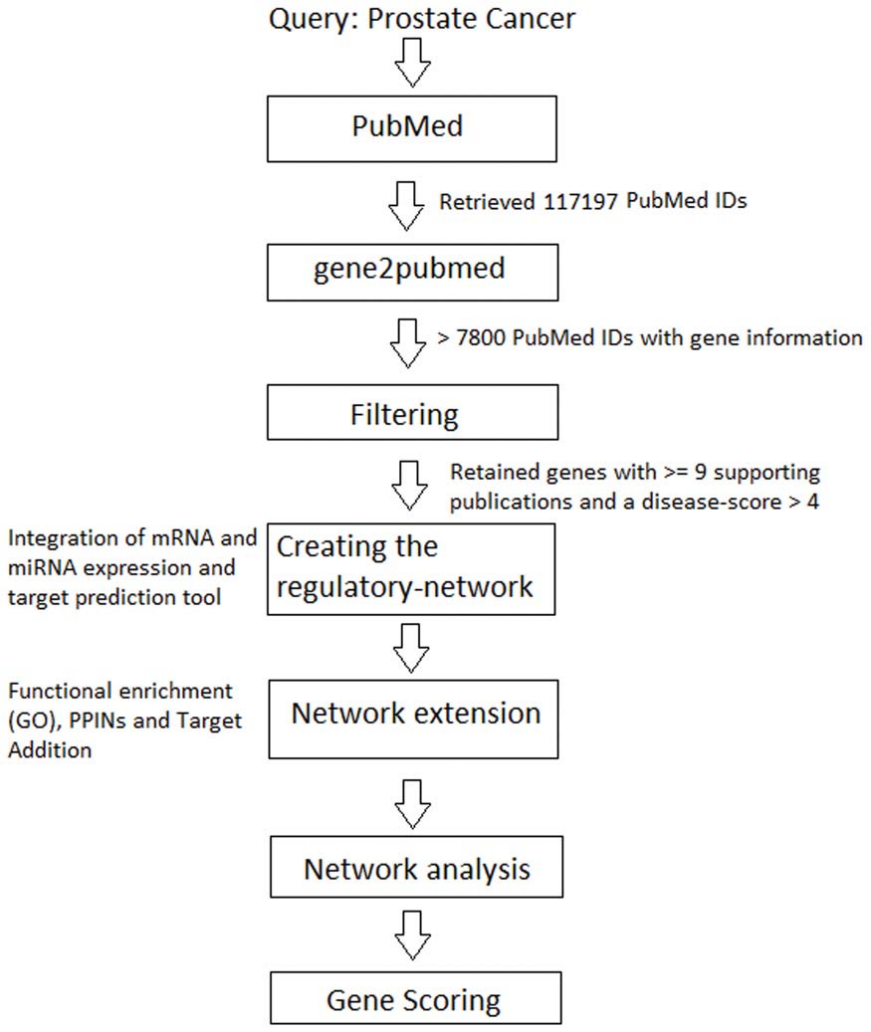
The *miRable* workflow. The method starts from a query to PubMed with the key words ‘prostate cancer’. We retrieved 117197 PubMed IDs and more than 7800 have gene information. We obtained 3757 gene IDs for human and of those, 250 genes have more than 9 supporting publications. 115 genes have a disease-score greater than 4.

In this section we provide a detailed description of the workflow of the *miRable* pipeline. As depicted in Fig 1, our method consists of three main steps: base regulatory network creation, network enrichment using pathology-specific data, and network analysis.

### Step One—Regulatory Network Creation

#### Literature Searching

Most often literature searching is the initial activity that researchers accomplish to identify a set of genes involved in a particular disease. Our method starts from this step and follows the approach proposed by Jourquin et al. [11] re-implementing it completely. In particular, we query PubMed (http://www.ncbi.nlm.nih.gov/pubmed) to retrieve a list of publications relevant to a particular pathology. The resulting list consists of a plethora of papers that, in most cases, do not contain references to genes. In order to focus only on the subset of articles containing gene information, we filtered the results according to the NCBI’s gene2pubmed database where the association between genes, PubMed papers and species is provided. This filtering ensures us to select only papers containing gene information. Once we have obtained a list of disease-related genes, we need a scoring strategy to give relevance to a gene with respect to all the others. To solve this issue we compute the hypergeometric score for all the retrieved genes, as Jourquin et al. [11] do, and we retain only the genes whose hypergeometric score (from now on referred to as *disease-score*) is higherthan 4. The disease score is computed by the following formula:

$$ds(m,n,j,k)=-log_{10}H(m,n,j,k)$$

where *H*(*m*, *n*, *j*, *k*) is computed by:

$$H(m,n,j,k)=\sum_{i=k}^{min(n,j)}\frac{\left(\begin{array}{c} m-j\\ n-i \end{array}\right)\left(\begin{array}{c} j\\ i \end{array}\right)}{\left(\begin{array}{c} m\\ n \end{array}\right)}$$

*m* is the number of articles restricted to homo sapiens’ genes, *n* is the number of papers regarding genes for homo sapiens related to the investigated pathology (in our case study prostate cancer), *j* is the number of articles about homo sapiens related to a certain gene *G*, and *k* is the number of papers about homo sapiens that are related to both the investigated pathology and *G*.

#### Exploiting miRNA-target predictions and gene expression

**Fig 2:**
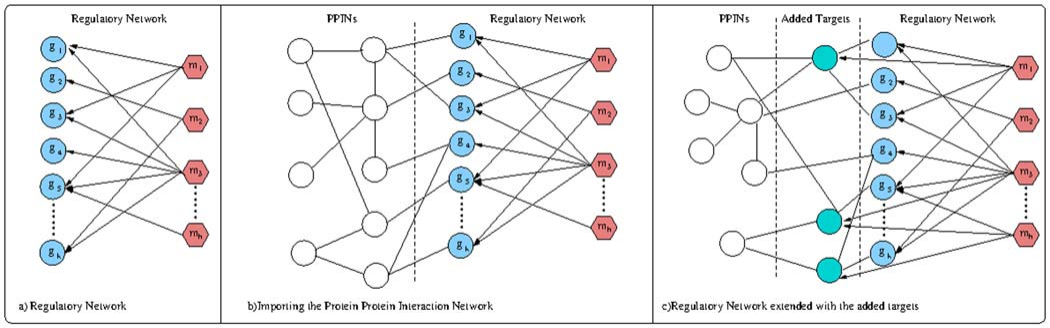
Modification of the regualtory network. a) Regulatory network. b) Regulatory network and PPINs. c) Extension of the regulatory network with new targets.

Once relevant genes have been selected mining the literature, they are required to be matched with the miRNAs for which they are targets to build the regulatory network, see Fig 2a). To accomplish this, a prediction step is applied on the retained genes, selecting all the miRNA-target pairs having as target one of the genes retained in the previous step. In this study we used TargetScan 6.2 [2] (the complete dataset with conserved and non-conserved binding sites) as miRNA-target prediction tool.

The normalized expression data comes from the Prostate Adenocarcinoma (MSKCC, Cancer Cell 2010) dataset obtained from cBioPortal (http://cbio.mskcc.org/cancergenomics/prostate/data/) [12]. This dataset consists of the complete mRNA (Affymetrix Human Exon 1.0 ST arrays) and miRNA (Agilent microRNA V2 arrays) expression profiles of 111 patients.

Not all the possible pairs are equally considered, because their importance also depends on the expression of the miRNAs and the targets in the specific context. Since miRNAs generally repress their target mRNAs, a natural way to validate mRNA targeted by a miRNA is that of measuring whether their expressions are inversely correlated. In fact, the value of the anti-correlation can be interpreted as a measure of the importance and strength of the miRNA-mRNA interaction. As a correlation coefficient we computed the Pearson’s score. The Pearson’s correlation coefficient is computed as $\rho_{XY}=\frac{\sigma_{XY}}{\sigma_{X}\sigma_{Y}}$ where *σ*_*XY*_ is the covariance of *X* and *Y*, *σ*_*X*_ is the standard deviation of *X*, *σ*_*Y*_ is the standard deviation of *Y*, *X* is the vector of the mRNA expression for each PCa tissue, and *Y* is the vector of the miRNA expression for each PCa tissue. We select the minimum absolute value such that the correlation is statistically significant (it depends on the number of samples in the expression dataset, and it is associated to a p-value ≤ 0.05) and we filter out all the pairs whose correlation coefficient is not high enough. The set *RC* of miRNA-target pairs retained can be defined as follows:

RC={(m,t)|m∈P(t),t∈GS(D),|CorrCoeff(m,t)|≥|min_corr|,CorrCoeff(m,t)<0}

where *GS(D)* is the subset of genes with a disease-score greater than 4 and *m* ∈ *P*(*t*) means that *m* is a miRNA predicted to target the mRNA *t*. The regulatory network consists of a bipartite graph whose nodes are all the miRNAs and targets in *RC* and there exists an edge connecting a miRNA-target pair if it belongs to *RC*.

### Step two—Regulatory Network Extension

Once the regulatory network is built, the goal is to extend it by predicting new pairs miRNA-mRNA that can be significant for the pathology, but never or poorly studied before. To this end we postulate that a gene has a higher probability to be associated to a specific pathology if it interacts with at least one of the known pathology-specific genes and also if it is involved in some functions highly relevant for the pathology.

#### Functional enrichment—pathology-relevant function retrieval

In this step our method associates to each gene in the regulatory-network the set of the Gene Ontology (GO) functions [13] in which the gene is involved. This information is subsequently used to select the subset of GO functions, called the Background Functions, that are more likely related to the pathology. The relevance of the function to the pathology is statistically determined by exploiting Fisher’s probability computation modified with Benjamini-Hockeberg’s method and selecting all the functions with *p* ≤ 0.05. The contingency matrix for the Fisher’s computation for each GO function *f* is as follows:

$$\begin{array}{c} \left|\begin{array}{cc} \#(f,\mathrm{GQ}) & \#(f,\mathrm{GGO})\\ \bar{\#(f,GQ)} & \bar{\#(f,GGO)} \end{array}\right| \end{array}$$

where #(*f*, *GQ*) is the number of genes considered in Step 1 that are involved in *f*, #(*f*, *GGO*) is the total number of genes involved in *f*, $\bar{\#(f,GQ)}$ is the number of genes considered in Step 1 not involved in *f*, and $\bar{\#(f,GGO)}$ is the total number of genes of the Gene Ontology that are not involved in *f*. To clarify this concept, let *m* be the number of genes in the GO, *n* be the number of genes returned in Step 1, *j* be the number of genes of the generic function *f*, and *k* ≤ *j* be the number of genes returned in Step 1 that are also involved in *f*. The contingency matrix then is:

$$\begin{array}{c} \left|\begin{array}{cc} k & j\\ n-k & m-j \end{array}\right| \end{array}$$

and the p-value for *f* is computed as: $Fisher(f,Q)=\frac{\left(\begin{array}{c} k+j\\ k \end{array}\right)\left(\begin{array}{c} n-k+m-j\\ n-k \end{array}\right)}{\left(\begin{array}{c} n+m\\ n \end{array}\right)}$.

The set of the Background Functions of a disease (*F*_*BF*_(*D*)) is defined as:

$$F_{BF}(D)=\{f\in GO|Fisher_{BH}(f,Q)\leq 0.05\}$$

where *Q* is the set of genes obtained from Step 1 and *Fisher*_*BH*_(*f*, *D*) is the *p-value* modified according to the Benjamini-Hockeberg method.

#### Network extension—importing the PPINs

Since all the genes belonging to the regulatory-network come from the literature, to add new targets not studied yet in conjunction with the pathology, we need to expand the set of genes. To achieve this, we add all the interactors of one mRNA by importing the subset of the Biogrid [14] Protein Protein Interaction network where the mRNA is an interactor. This procedure is repeated for each mRNA of the regulatory-network (see Fig 2 b) for a small example). The network is extended iteratively each time considering the interactors of the latest added mRNAs. The process stops when no more interactors can be imported. The output of this phase is an extended network that can be seen as a graph where each gene belongs at least to a path starting from a miRNA.

#### Target addition

According to the broadly accepted *guilt by association* principle, associated or interacting proteins are more likely to share functions. Hence, with the purpose of adding new potentially relevant targets in the regulatory-network, we focus on the subset of the imported mRNAs that are interactors of miRNA targets that is the set of previously imported genes having a direct connection with at least one miRNA target. These interactors are enriched with their GO functions. All the interactors having at least one GO background function are retained and considered as candidate targets. A candidate miRNA target is accepted if and only if: a) it is predicted by TargetScan as target for at least one miRNA belonging to the regulatory network; b) it is strongly anticorrelated (*p* − *value* ≤ 0.01) with respect to at least one of the miRNAs selected in step a). When a new mRNA verifies the two above conditions with a set of miRNAs *M*, it is included in the regulatory network as a new target and it is connected to all the miRNAs belonging to *M*. We repeat this procedure only once since a recursive application can result in the introduction of weak connections or misleading targets. Fig 2 c) provides an example of an extended regulatory-network. The complete list of miRNA-target pairs with their anticorrelation value can be found in the S1 File of this paper.

### Step three—Network analysis

#### Subnetworks extraction

**Fig 3:**
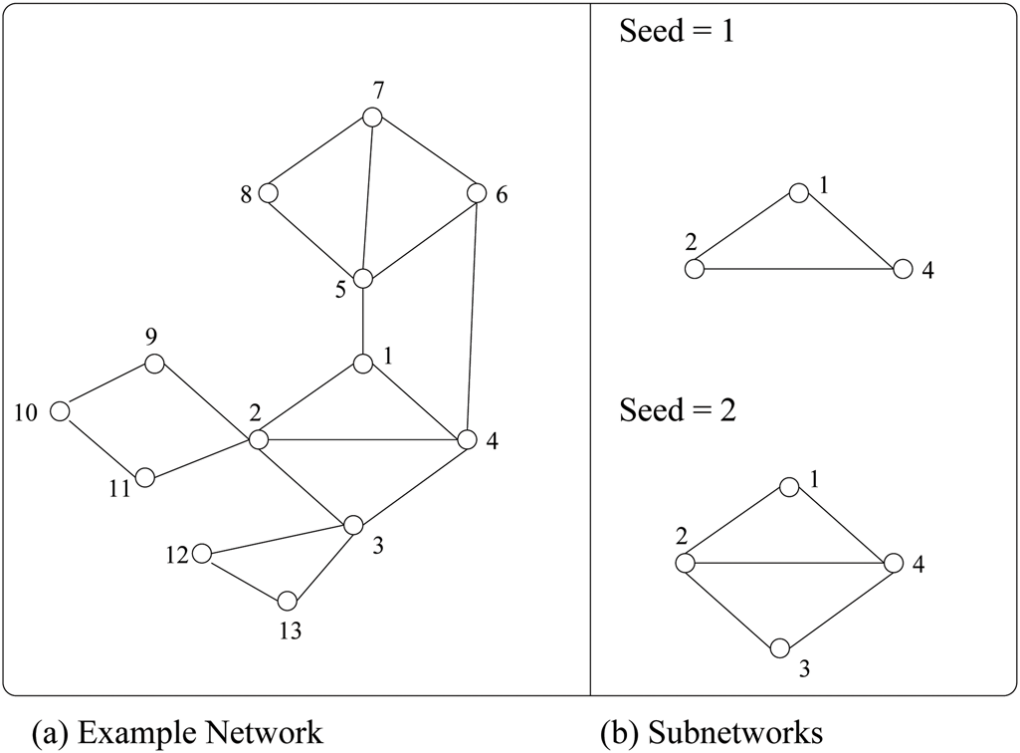
Construction of regulatory-subnetworks. a) Network toy example. b) Direct subnetworks with respect to two different seeds.

Starting from the idea that a pathology is the result of a complex set of interactions, and that a gene should always be considered not as a single entity, but as part of a context, we decided to explore the regulatory part of the network (that is the miRNAs and their direct targets after the target addition step) with the intent of extracting a subnetwork for each mRNA and for each miRNA. We consider each node (either mRNA or miRNA) of the regulatory network extended with the added targets, as a seed around which to build the subnetwork. Of this seed we take all the triangles. Consider for example the network in Fig 3 and node 1 as seed.

The neighbours of 1 is *N*(1) = {2, 4, 5}. So we consider all the neighbours of the seed neighbours without the seed itself: *N*(2) = {3, 4, 9, 11}, *N*(4) = {2, 3, 6}, and *N*(5) = {6, 7, 8}. Then we intersect the sets *N*(1) ∩ *N*(2) = {4}, *N*(1) ∩ *N*(4) = {2}, and *N*(1) ∩ *N*(5) = Φ, and we obtain the subnetwork of Fig 3b) up as subnetwork associated to the seed 1. The same Figure shows the subnetwork associated to seed 2. This procedure is iterated associating a subnetwork to each node of the extended regulatory network. The complete set of the extracted regulatory subnetworks can be found in the S2 File of this paper.

#### Centrality index computation

Since the regulatory subnetworks are direct unweighted graphs, it is possible to exploit standard measures derived from graph theory as tools to analyze them. Centrality indices [15] are among the basic and most frequently used graph theoretical measures to evaluate the importance of nodes within the network. Among the various centrality indices we computed: the betweenness centrality [16], the closeness centrality as defined in [17] where the index can be computed also on directed graphs, the eccentricity index [18], and the eigenvector centrality (a.k.a. power method index) [19]. According to [20] the power method index, of which we provide a brief definition below, is the most informative in cases like ours. Hence we decided to exploit mainly this index. The power method is an index that allows to measure the importance of the connections of a node by associating to each node a value that combines together its connections and their importance so that a node with a few connections of great importance can be more important than a node with many connections of low importance.

Let *x*_*i*_ the vertex *i* centrality, we define *x*_*i*_ as:

$$\begin{array}{c} x_{i}=\frac{1}{\lambda }\sum_{j=1}^{n}A_{ij}x_{j} \end{array}$$

According to the above formula, the centrality value of node *i* proportionally depends on the centrality of its neighbours and vice versa. As a result, computing centrality for a graph of *n* nodes reduces to the solution of a system of *n* equations in *n* unknowns. This problem can be efficiently solved by using an iterative approach. Let **x** = (*x*_1_, …, *x*_*n*_) be a vector where the *i*-th element contains the centrality of node *i*, then it is possible to compute **x** by solving the following equation: *λ* **x** = *A* **x** According to the above formula, **x** is the eigenvector of the adjacency matrix *A* with eigenvalue equal to *λ*.

#### Gene scoring

Result ranking is one of the most important things to consider to give advice about a pathology. In our case the best result is the gene showing the highest probability to be causative of the pathology. Hence, we ranked the genes using their regulatory subnetworks to obtain a score able to measure the impact of a gene in the disease. These subnetworks have the peculiarity that all their nodes are part of the regulatory network that is a node belongs to a regulatory subnetwork if it is contained in at least one intersection with the seed’s neighbour and it is also either a miRNA or neighbour of a miRNA. To compute this score (regulatory subnetwork score) we used a vector of p-values coming from the functional enrichment of the subnetwork. More precisely, for each function in the set of Background we compute the Fisher score for the function with respect to the subnetwork. The contingency matrix is built as seen before, but this time the set of considered genes are those belonging to the regulatory subnetworks and not those returned by the PubMed query. In this way we get a vector *v* of size *m* where *m* is the number of functions in *F*_*BF*_(*P*). To associate a single value with each vector of p-values, we took inspiration from Fisher’s combined probability test [21] and we computed the following score.

$$\begin{array}{c} S\left(v\right)=\sum_{i=1,v_{i}>0}^{m}-Log_{10}\left(v_{i}\right) \end{array}$$

We used this value to score the regulatory-subnetwork and so the seed it has started from: the highest the better.

We included in our method a further abstraction layer considering subnetworks as nodes of a meta-network where an edge is present if the seed of the edge target network belongs to the subnetwork of the edge starting network. This meta-network still maintains all the desirable theoretical properties of graphs and, thus, it can be analyzed with the same tools described earlier. In particular, we exploited the Power Method index as a ranking function so that to identify the most important subnetworks.

## Results and Discussion

In this study we proposed *miRable*, a new method aimed at exploring the complex world of miRNA regulation in the context of a disease. Starting from the literature information our method applies different constraints and filters to build the extended regulatory network, and exploits it to find all the regulatory subnetworks involved in a disease (in our case the PCa). By using this approach our method has been able to find new candidate genes and miRNAs related to PCa. Some of these genes have already been associated with PCa, but the majority of them are novel candidate genes.

In order to rank these genes according to their relevance for the analyzed pathology, we employ the regulatory subnetwork score. This score is able to capture the relevance of a regulatory subnetwork (and consequently its corresponding seed gene) for a disease on the base of the Background Functions. Moreover, we applied the Power Method on the meta network to estimate the importance of a seed in a regulatory subnetwork with respect to the other seeds. This index does not take into consideration the Background Functions associated to the disease, but only the seed’ connections. Both these scoring methods highlighted the same set of relevant best genes and miRNAs.

In our analysis we found that the most relevant genes in the ranking received few literature citations (see Table 1 and the S3 File), except for few cases such as the Androgen Receptor (AR).

**Table 1:** Top 20 genes in our result ranking list, based on the regulatory subnetwork score. A disease-score of -1,00 denotes a gene with less than 9 supporting publications related to PCa (at the time of the analysis) or no publications at all. RSs: regulatory subnetwork score; Ds: disease score; PMs: power method score.

**Gene**	**nr. nodes**	**RSs**	**Ds**	**PMs**
**EP300**	99	**1775.47**	0.10	0.12
**HDAC1**	103	1556.63	1.66	0.13
**CTNNB1**	78	1551.83	3.58	0.10
**ESR1**	154	1513.10	3.13	**0.19**
**AKT1**	89	1500.17	22.61	0.10
**AR**	69	1491.21	**768.47**	0.08
**SMAD3**	100	1464.57	0.68	0.12
**SP1**	64	1327.98	0.90	0.09
**hsa-miR-548c-3p**	151	1285.12	-1.00	0.11
**BRCA1**	142	1260.87	6.49	0.13
**MAPK8**	38	1232.48	0.91	0.05
**JUN**	56	1217.10	0.04	0.08
**KAT2B**	52	1187.84	-1.00	0.06
**STAT3**	53	1144.44	2.33	0.07
**CHUK**	39	1143.01	-1.00	0.06
**MYC**	135	1125.92	6.78	0.15
**hsa-miR-494**	147	1116.00	-1.00	0.13
**SIRT1**	62	1084.97	2.10	0.08
**RB1**	67	1053.97	0.69	0.08
**GSK3B**	62	1049.51	0.74	0.08

### Novel candidate PCa-related genes

By exploiting the two measures (the regulatory subnetwork score and the Power Method score) described in the previous section, our method predicted some genes that have already been studied (i.e. EP300, CTNNB1, AKT1, AR), while others are less known in relation to PCa. In the following, we provide some details about three promising predicted genes in the top rank positions: ESR1, miR-548c-3p and miR-494.

**Fig 4:**
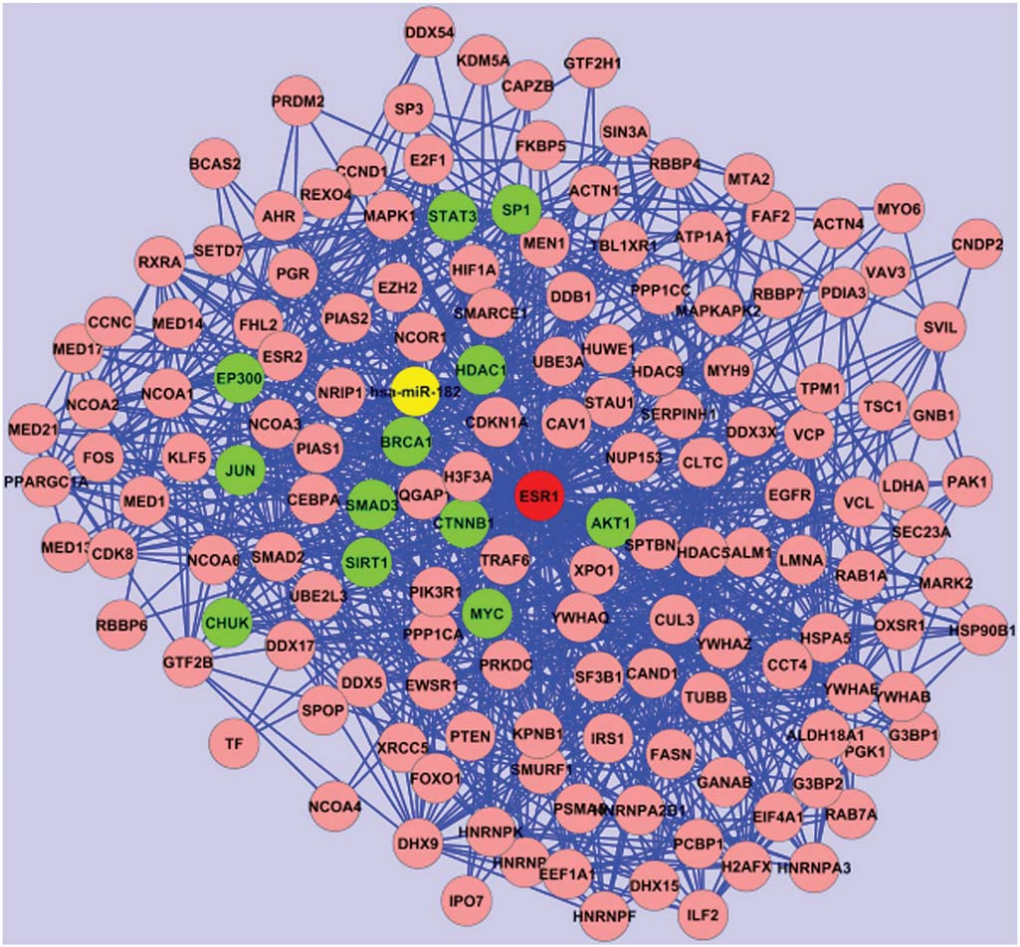
ESR1 regulatory subnetwork. Red node: ESR1; yellow node: miR-182; green nodes: genes in our top 20 ranking list (EP300, CTNNB1, AKT1, BRCA1, SMAD3, HDAC1, MYC, SP1, STAT3, CHUK, SIRT1, JUN). Cytoscape (http://www.cytoscape.org/) has been used to visualize the network.

**Fig 5:**
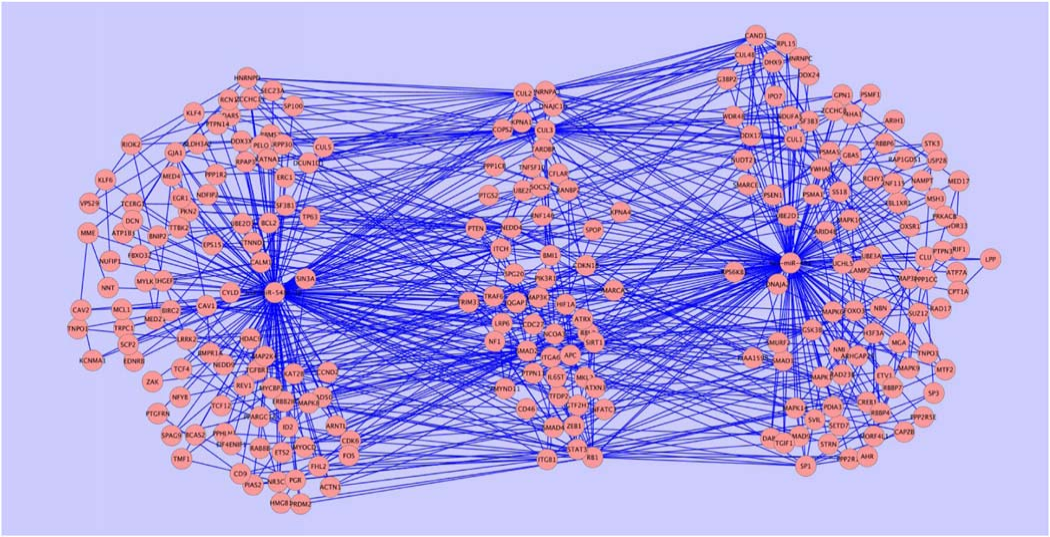
Regulatory-subnetworks for miR-494 and miR-548c-3p. On the left the number of targets of miR-548c-3p in its regulatory subnetwork; on the rigth the number of targets of miR-494 in its regulatory subnetwork. In the middle the common targets of both miRNAs.

**Fig 6:**
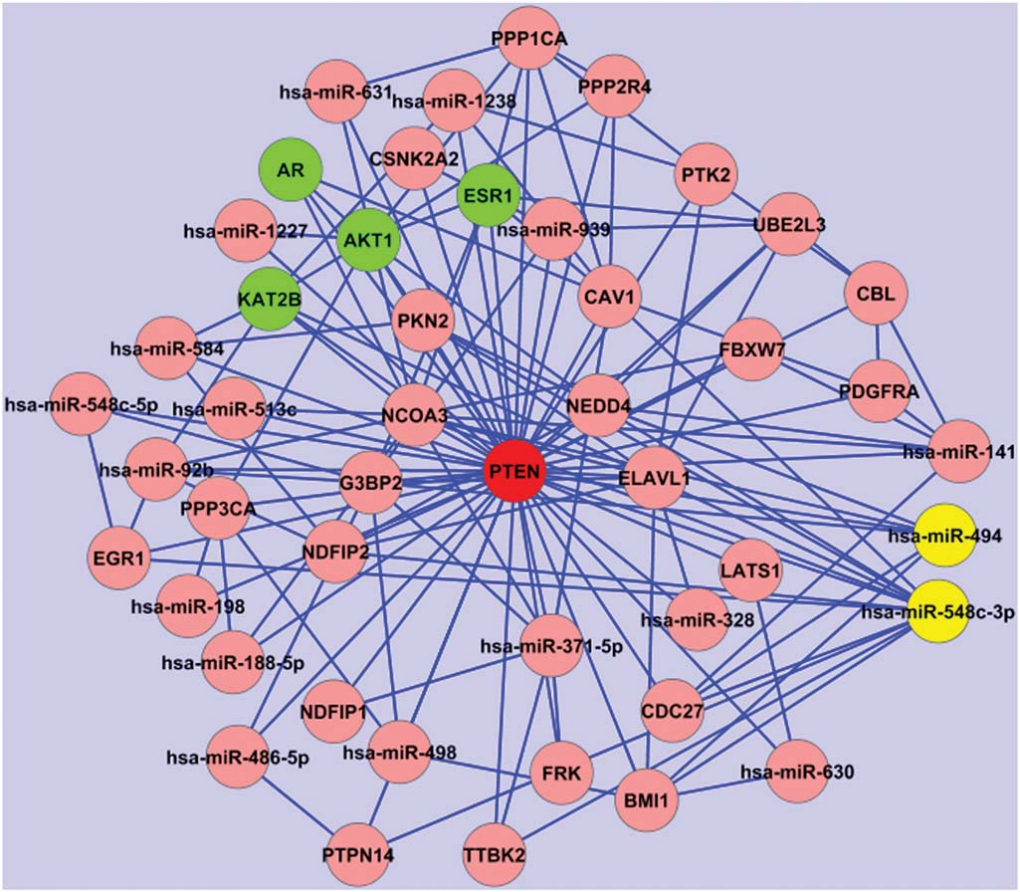
PTEN regulatory subnetwork. Red node: PTEN; yellow nodes: miR-548c-3p and miR-494; green nodes: genes in the top 20 ranking list (ESR1, AR, AKT1, KAT2B). Cytoscape (http://www.cytoscape.org/) has been used to visualize the network.

- **Estrogen Receptor 1 (ESR1)**. The Estrogen Receptor 1 is an inhibitor of cell migration and its repression enhances cell migration and accelerates tumor formation and metastasis [22]. This gene is known to be involved in breast cancer. Recent publications focused on ESR1 polymorphisms to explore potential associations with prostate cancer risk. In a recent work Fu et al. [23] conducted a meta-analysis indicating that ESR1 rs9340799 polymorphism is associated with prostate cancer risk. According to our results ESR1 is among the top ranked genes (see Table 1 and Fig 4).
  Even though its disease-score, based on publications, is only 3.13, its regulatory subnetwork score is 1513.10 with 154 nodes in the subnetwork and the highest Power Method score (0.19). Interestingly, although the ESR1 disease-score is below the defined threshold of 4 and thus it is removed in the first-phase filtering, our method re-included this gene into the regulatory network in the second step of our algorithm (namely the regulatory-network extension), demonstrating the efficacy of the method to discover unstudied or poorly studied potentially-relevant disease-related genes.
  By using our method we found that ESR1 is a potential candidate target of miR-182. This miRNA is over-expressed in PCa tissues and cell lines and it has been shown that it can promote cell invasion and proliferation, and knock-down of miR-182 also significantly decreased in vivo prostate tumor growth [24]. Hence, we can conclude that the over-expression of miR-182 and the down-regulation of ESR1 could be associated with PCa progression and potentially useful as prognostic biomarker. Moreover, Tang and colleagues [22] have recently applied a computational approach identifying two putative novel miRNA regulatory pathways in PCa: 1) ligand-independent activation of ESR1 and ESR2 and 2) membrane-bound ESR1 (interaction with growth factors signalling), showing a new possible role of miRNAs in the regulation of ESR1 and the consistence of our results.
  It is interesting to note that ESR1 and the other genes in our top 20 list interact with each other. Among these, ESR1 interacts with: EP300, CTNNB1, AKT1, BRCA1, SMAD3, HDAC1, MYC, SP1, STAT3, CHUK, SIRT1, JUN. Some of these genes have already been reported to be abnormally expressed in prostate cancer, such as for example the Histone deacetylase 1 (HDAC1) [25] which is in the second position in the ranking list, based on the regulatory subnetwork score.
- **miR-494 and miR-548c-3p.** According to our results, miR-494 and miR-548c-3p are the top rank candidate miRNAs involved in PCa. This result is based on both: the number of interactions (respectively 146 for miR-494 and 150 for miR-548c-3p), the regulatory subnetwork score (1116.00 for miR-494 and 1285.12 for miR-548c-3p) and their Power Method score (see Table 1). Nevertheless, the role of both these miRNAs in PCa is poorly known.
  Little is known about miR-548c-3p while more information about the miR-548 family is available. The latter is a large, poorly conserved primate-specific miRNA gene family [26]. It has been postulated that the miR-548 family could derive from Made1 transposable elements [27]. Made1 elements are short miniature inverted-repeat transposable elements (MITEs), which consist of two 37 base pair (bp) terminal inverted repeats that flank 6 bp of internal sequence. Thus, Made1 elements are nearly perfect palindromes, and when expressed as RNA they form highly stable hairpin loops. The role of miR-548 family in cancer is still to be elucidated, but some authors suggested cancer-related regulatory roles for this family [27].
  The role of miR-494 is better known. In fact, several publications discuss its role in cancer [28] [29] [30]. Liu et al. [28] identified miR-494, whose expression is induced by tumor-derived factors, as an essential player in regulating the accumulation and activity of Myeloid-Derived Suppressor Cells (MDSCs). These MDSCs orchestrate the tumor microenvironment and facilitate tumor angiogenesis and metastasis, by direct targeting of PTEN and activation of the Akt pathway.
  As shown in Fig 5, miR-494 and miR-548c-3p regulatory subnetworks share 54 putative targets including PTEN. Observing PTEN regulatory subnetwork (Fig 6), we can see 47 nodes, of these 18 miRNAs (two of them, miR-494 and miR-141, have already been validated [31]), and other important genes in our ranking list such as: ESR1, AR, AKT1, KAT2B.
  It has been shown that miR-141 is up-regulated in PCa tissues and cell lines and it modulates androgen receptor transcriptional activity in human PCa cells [32]. Moreover, according to the miRandola database [33, 34], it is considered a new candidate non-invasive biomarker because it is frequently over-expressed in the blood of PCa patients compared to healthy controls.

## Conclusions

In this work we presented a new method aimed at exploring the complexity of gene regulation through miRNAs, in a disease-specific context. Our method allows to: 1) find new potential miRNA-targets, 2) highlight novel genes potentially involved in a disease but never or poorly studied before, 3) reconstruct all possible regulatory subnetworks starting from the literature to expand the knowledge on the miRNA regulatory mechanisms.

We applied our method to prostate cancer finding new interesting protein-coding genes and miRNAs potentially involved in the disease. The most promising protein-coding candidate gene is ESR1. Its role is well-understood in breast cancer, instead its role in prostate cancer has not been investigated adequately. Nevertheless, a number of similarities between the AR and ESR1 signalling pathways are known. In the absence of a ligand they are inactive and complexed with heat shock proteins [35]. When the ligand (androgen or estrogen) binds the receptor, the receptor itself undergoes to a conformational change promoting its nuclear localisation, dimerization and DNA binding. Furthermore, AR and ESR1 recruit common cofactors for both pathways.

Considering the above similarities between breast cancer and prostate cancer, as a future work we plan to test our method on breast cancer to understand whether there are common relevant protein coding genes and miRNAs in our top ranking list thus defining more possible common pathways. Moreover, we plan to integrate other regulators of gene expression (e.g. transcription factors) to find more complex regulatory networks and circuits.

## Supporting Information

S1 File Mirna-target interaction. This is a csv file containing the list of all the miRNA—target interactions considered, their anti correlation value and an indicator stating if the miRNA-target pair is validated.
(CSV)

S2 File Regulatory subnetworks. This file contains all the discovered regulatory subnetworks.
(ZIP)

S3 File Scoring list. This is a csv file containing the complete ranking list related to Table 1.
(CSV)
